# Oxidative Stress in Diabetes: Implications for Vascular and Other Complications

**DOI:** 10.3390/ijms141121525

**Published:** 2013-10-30

**Authors:** Dario Pitocco, Manfredi Tesauro, Rizzi Alessandro, Giovanni Ghirlanda, Carmine Cardillo

**Affiliations:** 1 Department of Internal Medicine, Catholic University Medical School, Largo Gemelli 8, Rome 00168, Italy; E-Mails: dario.pitocco@rm.unicatt.it (D.P.); alessandro.rizzi.86@gmail.com (R.A.); gghirlanda@rm.unicatt.it (G.G.); 2 Department of Internal Medicine, University of Rome “Tor Vergata”, Viale Oxford 81, Rome 00133, Italy; E-Mail: mtesauro@tiscali.it

**Keywords:** oxidative stress, reactive oxidative species, diabetes mellitus, insulin resistance, β-cell dysfunction, vascular complications

## Abstract

In recent decades, oxidative stress has become a focus of interest in most biomedical disciplines and many types of clinical research. Increasing evidence shows that oxidative stress is associated with the pathogenesis of diabetes, obesity, cancer, ageing, inflammation, neurodegenerative disorders, hypertension, apoptosis, cardiovascular diseases, and heart failure. Based on these studies, an emerging concept is that oxidative stress is the “final common pathway” through which the risk factors for several diseases exert their deleterious effects. Oxidative stress causes a complex dysregulation of cell metabolism and cell–cell homeostasis; in particular, oxidative stress plays a key role in the pathogenesis of insulin resistance and β-cell dysfunction. These are the two most relevant mechanisms in the pathophysiology of type 2 diabetes and its vascular complications, the leading cause of death in diabetic patients.

## 1. Introduction

The biological systems living in aerobic conditions are exposed to oxidants, either generated intentionally or as byproducts. Generally, these oxidants occur in two categories consisting of paramagnetic free radicals: reactive oxygen species (ROS) and reactive nitrogen species (RNS). ROS is a collective term used to describe the chemical species that are formed upon incomplete reduction of oxygen and includes superoxide anion (O_2_^−^), hydrogen peroxide (H_2_O_2_), and hydroxyl radical (HO^•^). In contrast, RNS refers to all the oxidation states and reactive adducts of nitrogenous nitric oxide synthase (NOS) products, from nitric oxide (NO) to nitroxyl (NO^−^), *S*-nitrosothiol (RSNO), and peroxynitrite (OONO^−^) [1]. ROS and RNS, previously considered to be toxic agents capable to damage molecules, have indeed critical biological functions essential for normal physiology. All these species are able to initiate or mediate many enzyme- and gene-dependent reactions in both physiological and pathophysiological processes. Overproduction or deficiency of ROS and/or RNS may result in impaired homeostasis and associated pathology. Thus, it is widely believed that multiple pathogenic mechanisms involve disequilibrium in the redox balance as the final common pathway [1].

### 1.1. Methods for Detecting Oxidative Stress Metabolites

In clinical practice, analytical measurement of oxidative stress markers has been difficult. The difficulties are due to the short half-life of the majority of these compounds (few seconds) and to the applicability of the determination methods. Blood and urine samples are the eligible biological materials for assessment of the oxidant and antioxidant status in humans; however, some antioxidant enzymes and metabolites of oxidative stress have also been determined in tissue extracts, especially in animal models [2,3]. A variety of methods, including enzyme-linked immunosorbent assay (ELISA), high pressure liquid chromatography (HPLC), spectroscopy, gas chromatography-mass spectometry (GC-MS), immunoblotting, electroelution fractionation, isoelectric focusing, voltammetry, and electron paramagnetic resonance (EPR) spectroscopy have been applied for determination of metabolites of oxidative stress [4–6]. A dynamic indicator of oxidative stress *in vivo* is the quantification of the redox state of glutathione (GSH/GSSG) in tissues and/or plasma. This can be determined biochemically [4] or by HPLC according to the method described by Jones [7]. Electron spin resonance (ESR) spectroscopy, also known as electron paramagnetic resonance (EPR) spectroscopy, is the only analytical approach that enables direct detection of free radicals such as NO, superoxide, and hydroxyl radical [8]. With its limited sensitivity of 10^−9^ M, it is capable of detecting free radical-derived species produced during oxidative and inflammatory injury [9]. Other methods have been developed to indirectly detect oxidant/free radical formation *in vitro* and *in vivo*. Lipid peroxidation is one of the most widely used indicators for determining the cellular oxidant status [10]. HPLC and several commercially available ELISA kits have been developed to quantify other attractive indicators of lipid peroxidation, such as F2-isoprostanes [11,12]. Another method for measuring reactive oxygen metabolites (ROM) in blood is the so-called d-ROM test, based on a free radical analytical system that determines serum lipid hydroperoxides, the main component of ROM [13]. Given its relative stability, hydroperoxide is relatively stable and easily detectable. Laboratory measurement of NO, by contrast, is extremely difficult because of its short half-life and its very low concentrations in biological fluids. Nitrite (NO_2_^−^) and nitrate (NO_3_^−^) are the most stable metabolites of endogenous NO. They are accessible by quantitative analysis, so that determination of these inorganic metabolites in blood and urine turned out to be the most suitable method to indirectly assess NO production *in vivo*. Mass spectrometry-based methods are currently the most accurate for quantitative determination of nitrate and nitrite in biological fluids. Also other analytical methods, as gas and liquid chromatography and electrophoresis, have been applied [14–18]. Commercially available kits are based on the Griess reaction that allows spectrophotometric detection of nitrate and nitrite. Due to their simplicity, rapidity and cheapness, they have been broadly applied in clinical practice. However many pre-analytical and analytical factors can interfere with this reaction: protein content of biological samples, turbidity related to aggregation or precipitation of proteins, conversion of nitrite to *S*-nitroso compounds by reduced thiols, and other reactions with tyrosine and tryptophan. An appropriate de-proteinization procedure of the serum samples, can be successfully applied for measurement of NO metabolites [19,20]. Thus, nitrite-plus-nitrate (NO*_x_*) levels measured by this procedure are in keeping with those detected by either CG-MS or HPLC [21]. Increasing interest exists in investigating the correlation between NO production and asymmetric dimethylarginine (ADMA), an endogenous nitric oxide synthase inhibitor. The existing methods for ADMA determination are HPLC, liquid chromatography-mass spectrometry (LC-MS) and GC-MS [22]. More recently, an ELISA kit for detection of ADMA has been evaluated against reversed phase HPLC and, despite the differences between the two methods, they have shown a good correlation [23]. Antioxidant capacity (AOC) includes endogenous compounds like bilirubin, uric acid, superoxide dismutases, catalase, and glutathione peroxidase, as well as exogenous substances, such as carotenoids, tocopherols, ascorbate, and bioflavonoids [24]. These antioxidant molecules are subdivided into three categories: enzyme systems, small molecules and proteins. Total antioxidant capacity (TAC) measures either the combination of both small molecule antioxidants and proteins or small molecules alone. An enhanced chemiluminescence assay is commonly used to measure the non-enzymatic TAC, but it is expensive and time-consuming; therefore, commercial colorimetric kits are frequently preferred [25]. Finally, most of the serum AOC-related indirect biochemical markers, such as bilirubin, total cholesterol, low-density lipoprotein cholesterol, high-density lipoprotein cholesterol, triglyceride, alanine aminotransferase, blood urea nitrogen, iron, and uric acid are commonly measured by well-established routine procedures based on automated systems.

### 1.2. ROS and RNS in Physiology and Pathophysiology

Production of ROS and RNS occurs in response to extracellular and intracellular stimuli. Extracellular stimuli act through plasma membrane receptors and include tumor necrosis factor (TNF)-α, hormones and growth factors, including platelet-derived growth factor, epithelial growth factor and insulin. Intracellular stimuli include nutrients [26,27], nicotinamide adenine dinucleotide phosphate (NADPH) oxidase [28], NOS [29], mitochondrial electron transfer [30], enzymatic systems in which ROS are generated (lipoxygenase, xanthine oxidase) and detoxification mechanisms (P-450 system) [31]. These are the most relevant sources of reactive species (RS), including ROS and RNS. They can react with multiple cellular components (proteins, lipids, nucleic acids) to generate reversible or irreversible oxidative modifications. They also activate various signaling cascades designated for sensing and responding to “stress”, such as the mitogen-activated protein (MAP) kinase family, as well as that of the c-Jun *N*-terminal kinase (JNK) [32]. The functional effects of RS range from physiology to pathophysiology: vascular tone [33], cell adhesion [34], immune responses [35], cell growth and hormone action [36] are all examples of RS participation in normal physiology; on the other hand, a causative role of RS has been involved in ageing [37], cancer [38], atherosclerosis [39], neurodegenerative diseases [40], obesity and diabetes [41]. It is important, therefore, to understand how ROS signaling in normal physiological processes can be transformed into damaging enzyme-catalyzed pathways. It is thought that RS contribution to normal physiology involves their carefully regulated production in a tight spatiotemporal manner, hence leading to reversible oxidative modifications. The pathophysiological processes mediated by RS, by contrast, are more likely to involve irreversible modifications of cellular components, such as proteins, lipids, or DNA.

## 2. Reactive Oxygen Species in Diabetes Mellitus

There is a bulk of evidence demonstrating that mitochondrial ROS (predominantly superoxide anion) overproduction is involved in diabetes and diabetic complications, even though it is difficult to identify the exact site of ROS formation in the mitochondria. Earlier work suggested that glucose can directly stimulates ROS overproduction [42], but it was later shown that high glucose (HG) activates various enzymatic cascades in mitochondria, including activation of NADPH oxidase, uncoupling of NO synthases and stimulation of xanthine oxidase [42–46]. Glycated proteins can also be the promoters of ROS formation [44], thus suggesting that different sources may be responsible for ROS overproduction and oxidative stress in diabetes. The exact role of mitochondria is not completely clear. For some time, mitochondria have been considered the major source of ROS in diabetes and diabetic complications, but Martens *et al.* have demonstrated that HG might actually suppress mitochondrial superoxide formation in metabolically responsive pancreatic β-cells [47]. Similarly, Herlein *et al.* have shown that there is no excess of superoxide production by complexes I and III from mitochondria of streptozotocin diabetic rats [48]. In addition, Hou *et al.* have reported significant ROS generation under low glucose conditions in mouse β-cells, which is prevented by the ROS scavengers *N*-acetylcysteine (NAC) and manganese(III)tetrakis(4-benzoic acid) porphyrin [49]. Other studies assert an increase of the number of mitochondria. Although their role seems to be controversial, mitochondria are the main source of ROS and further studies are required to deeply analyze their action.

### 2.1. Oxidative Stress, β-Cells and Insulin Secretion

Oxidative stress with ensuing glucotoxicity and lipotoxicity are diabetes-related phenomena that have been involved in the pathogenesis of β-cell dysfunction [50]. Thus, hyperglycemia and hyperlipidemia that follow the primary pathogenic process of diabetes may exert additional toxic effects on β-cells. Evidence resulting from *in vitro* and *in vivo* studies suggests that both glucose and lipids are indeed harmful for the β-cells. Interestingly, some studies have reported that lipotoxicity only occurs in the presence of concomitantly elevated glucose levels [51,52]. Consequently, hyperglycemia might be a prerequisite for the negative effects of lipotoxicity, hence the term glucolipotoxicity may be preferred to lipotoxicity to better describe the harmful relationship between lipids and β-cell function. Some authors have demonstrated that insulin gene expression, insulin content, and glucose-induced insulin secretion are progressively and drastically compromised over time when β-cell lines (HIT-T15 cells) are exposed to high glucose concentrations [53].

Decreased levels of insulin mRNA, insulin content, and insulin release have been regarded as evidence of the glucotoxic effects on β-cells of chronic exposure to high glucose (HG) concentrations. Evidence that glucotoxicity is related to oxidative stress stems from early reports showing that antioxidants, such as *N*-acetylcysteine and aminoguanidine, protect HIT-T15 cells and isolated islets against the adverse effects of exposure to high glucose concentrations [54,55]. Use of antioxidants, such as *N*-acetylcysteine or aminoguanidine, in cells cultured for many passages under high glucose conditions allows the detection of concentration-related preservation of both insulin promoter activity and insulin mRNA [56]. A prolonged *in vitro* exposure of isolated islets or insulin-secreting cells to elevated levels of fatty acids, by contrast, is associated with inhibition of glucose-induced insulin secretion, impairment of insulin gene expression, and induction of cell death through apoptosis. Of note, most of these glucolipotoxicity-related effects on β-cells involve generation of oxidative stress and inflammation. Furthermore, pancreatic β-cells exposed to hyperglycemia may produce ROS, which, in turn, suppress glucose-induced insulin secretion (GIIS). In fact, Sakai *et al.* observed that high glucose induced mitochondrial ROS suppress the first-phase of GIIS through inhibition of glyceraldehyde 3-phosphate dehydrogenase (GAPDH) activity [57]. Krauss *et al.* demonstrated that endogenously produced mitochondrial superoxide activates uncoupling protein 2 (UCP2)-mediated proton leak, thus leading to lower ATP levels and impaired GIIS [58]. Pi *et al.* reported that β-cells have relatively low expression of many antioxidant enzymes, making these cells susceptible to ROS-induced damage; at the same time, however, HG-induced ROS signaling may stimulate insulin secretion, thus suggesting that insulin secretion may be stimulated by HG-induced H_2_O_2_[59]. The latter finding is strengthened by the observation of Leloup *et al.*, who showed that mitochondrial ROS production is necessary for glucose-induced insulin secretion [60]. By contrast, heightened oxidative stress may be deleterious for insulin secretion, as suggested by the demonstration that the secretory response to glucose is reduced by 40% in rat islets or cells stressed for 3 days [61] and that ROS-mediated GIIS may further increase ROS production [62].

### 2.2. Oxidative Stress and Insulin Signal Transduction

Under physiological conditions, insulin signals through a signaling cascade that includes insulin or insulin-like growth factor (IGF)-1, insulin receptor (IR), insulin receptor substrate (IRS)-1 and phosphatidylinositol-3 kinase (PI3-K)/Akt or ERK kinases. ROS overproduction can disturb this process at different stages, consequently resulting in either insulin resistance or enhanced insulin signal. High concentrations of H_2_O_2_ activate insulin signaling and induce the typical metabolic actions of insulin [63] by causing downstream propagation of its signal. H_2_O_2_, therefore, increases glucose uptake in adipocytes and muscle cells [64] and stimulates GLUT4 translocation and lipid biosynthesis in adipocytes [65]. IRS-1, the effector of tyrosine kinase activity of the IR upon insulin binding, is involved in a critical step of insulin signaling. Under normal states, insulin signaling molecules are distributed between cytosol and internal membrane pools; following insulin stimulation, tyrosine residues on IR and IRS are phosphorylated by activated insulin receptor kinase. This leads to the recruitment of PI3-K to the plasma membrane and the internal membrane pools. Subsequently, activation of the small GTPase Rac induces a reorganization of the cytoskeleton that propagates the insulin signal and finally leads to increased glucose uptake. Under conditions of increased oxidative stress, however, stress-responsive signaling cascades, such as the MAP kinase, are activated, leading to increased Ser/Thr phosphorylation of IRS molecules. Modified IRS molecules are released from the internal membrane pools and undergo enhanced protein degradation. Under these conditions, insulin fails to elicit its normal metabolic effects, because IRS molecules content is decreased and cannot be tyrosine phosphorylated due to hyperphosphorylation of certain Ser/Thr residues [66].

Mitochondria can be a source of TNF-α-induced ROS production in cells and this may contribute to the pathogenesis of TNF-α-induced insulin resistance. It has been shown that TNF-α-stimulated mitochondrial production of ROS induces apoptosis signal-regulating kinase-1 (ASK-1) and activates JNK, thereby increasing Ser-307 phosphorylation of IRS-1 and decreasing insulin-stimulated tyrosine phosphorylation [67]. Chronic treatment of 3T3-L1 adipocytes with TNF-α activates intracellular IKKβ and reduces tyrosine phosphorylation of IRS-1, ultimately leading to impaired insulin action. Obese rodents treated with a TNF-α neutralizing antibody exhibit reduced hyperinsulinemia [68]. Whole body deletion of TNF-α or its corresponding receptor *TNF receptor 1* (*TNFR1*) gene partially protects mice from obesity-induced IR [69]. Increased production of TNF-α has also been widely associated with obesity-related insulin resistance and abnormal vascular reactivity, the vasculature being an important target of TNF-α [70]. Indeed, circulating TNF-α may impair vascular function by altering the balance between endothelial-derived vasodilator and vasoconstrictor substances because it downregulates the expression of eNOS [71] and upregulates ET-1 production in endothelial cells [72]. It may also directly activate NAD(P)H oxidase and increase the production of reactive oxygen species (ROS) in endothelial and vascular smooth muscle cells [73]. Furthermore, adipose tissue-derived TNF-α may suppress insulin-mediated hemodynamic and metabolic effects through inhibition of IRS-1 phosphorylation [74]. In addition to these direct effects on the vasculature, TNF-α might induce vascular dysfunction indirectly through stimulation of lipolysis, hence resulting in increased release of non-esterified fatty acids (NEFA). In healthy humans, infusion of TNF-α inhibits insulin’s stimulating effect on glucose uptake and endothelium-dependent vasodilation [75]. Conversely, a study performed in obese women has demonstrated that weight loss over 1 year results in a significant reduction in circulating TNF-α levels with parallel amelioration of endothelial function [76]. In our laboratory, we tested the possible role of TNF-α in vascular dysfunction associated with the metabolic syndrome by use of the TNF-α neutralizing antibody infliximab. We observed that infliximab enhances the stimulatory effects of insulin on both endothelium-dependent and -independent vasodilator activity [77]. The effect of TNF-α blockade on vascular function was not additive to that observed after administration of the antioxidant vitamin C, hence suggesting that TNF-α-related vascular dysfunction in the human vasculature is associated with increased oxidative stress [78].

In addition to vascular protection, TNF-α antagonism has also proven effective in protection against other diabetic complications in experimental models. Thus, TNF-α inhibition decreased urinary albumin excretion in diabetic rats, as indicated by the reduction in 24 h urinary albumin/creatinine ratio (U_alb_/U_cr_), thereby suggesting that it might be a potential therapeutic strategy for diabetic nephropathy [79]. In addition, in rats with streptozocin-induced diabetic cardiomyopathy, TNF-α neutralizing antibody improved left ventricular function by reducing myocardial inflammation and fibrosis [80].

### 2.3. Oxidative Stress and Insulin Resistance

**Figure 1:**
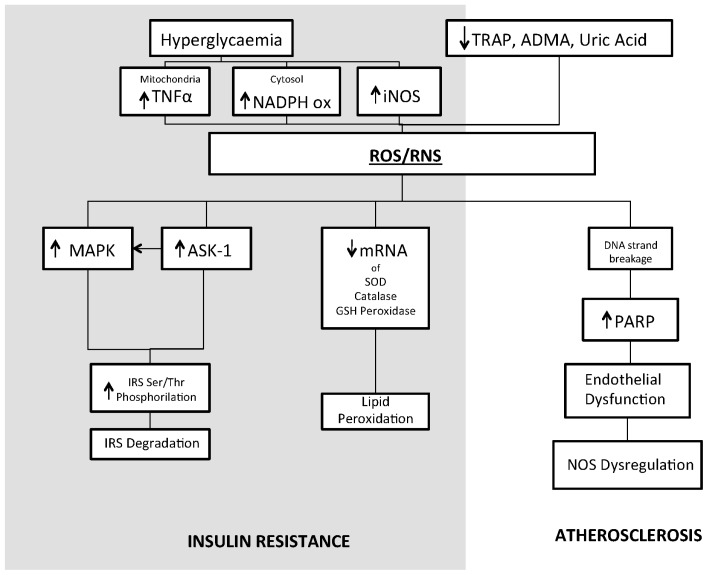
Proposed mechanisms by which increased oxidative stress in diabetes may lead to insulin resistance and atherosclerosis. All abbreviations are spelled out in the text. ↑ indicates increased levels; ↓ indicates decreased levels.

A large number of studies have provided evidence for the pivotal role of oxidative stress in insulin resistant states such as obesity, the metabolic syndrome and type 2 diabetes [81–84]. Thus, ROS overproduction is an important trigger for insulin resistance and a relevant factor in the development of type 2 diabetes [85] (Figure 1). Again, mitochondria and NADPH oxidase are considered the major sources of ROS overproduction, given that mitochondrial superoxide production is a common feature in models of insulin resistance in adipocytes, myotubes and mice. Several animal studies have been performed to investigate the role of increased oxidative stress in insulin resistant states. In obese mice, increased H_2_O_2_ generation by adipose tissue can be observed prior to the onset of diabetes [86]. This event is accompanied by decreased mRNA levels of SOD, catalase and glutathione peroxidase and all these changes are exaggerated by the development of diabetes. Obesity and related insulin resistance are frequently associated with increased accumulation of lipids (triglycerides) in the liver. Increased lipid peroxidation markers have thus been observed in the liver of animal models of diabetes and obesity [87]. Evidence of systemic oxidative stress includes detection of increased circulating and urinary levels of the lipid peroxidation product F2-isoprostane (8-epi-prostaglandin F2α) in both types 1 and 2 diabetes [88,89], as well as in obesity. Remarkably, this marker correlates with blood glucose levels and glucose variability, and ameliorates following therapeutic interventions [90].

### 2.4. Antioxidant Deficiency

In addition to overproduction of oxidant agents, higher oxidative stress may be due to the reduction of plasma antioxidant capacity. Even though Savu *et al.* have reported higher levels of antioxidants in patients with uncomplicated type 2 diabetes [91], a number of studies have shown a reduction of plasma antioxidant capacity occurring already even in the early phase of the disease. Thus, patients with type 1 diabetes of recent onset present a lower level of total radical antioxidant products (TRAP) compared to healthy people [92], which can be detected at the moment of the first diagnosis before the development of complications. Another well recognized antioxidant agent is uric acid, which plays its role in two different ways: it promotes superoxide dismutase activity and enhances the action of ascorbate. Lower level of blood and urinary uric acid have been detected in women with type 1 diabetes, in whom uric acid reduction was associated with increased oxidative stress [93]. Oxidation-induced alterations in molecules involved in insulin signaling are also associated with impaired insulin action, as shown in a rat model of oxidative stress induced by inhibition of glutathione biosynthesis. In this model, the drop in tissue levels of glutathione, a major cellular antioxidant, was associated with increased oxidative stress and impaired glucose homeostasis [41].

### 2.5. Oxidative Stress and Vascular Damage

**Figure 2:**
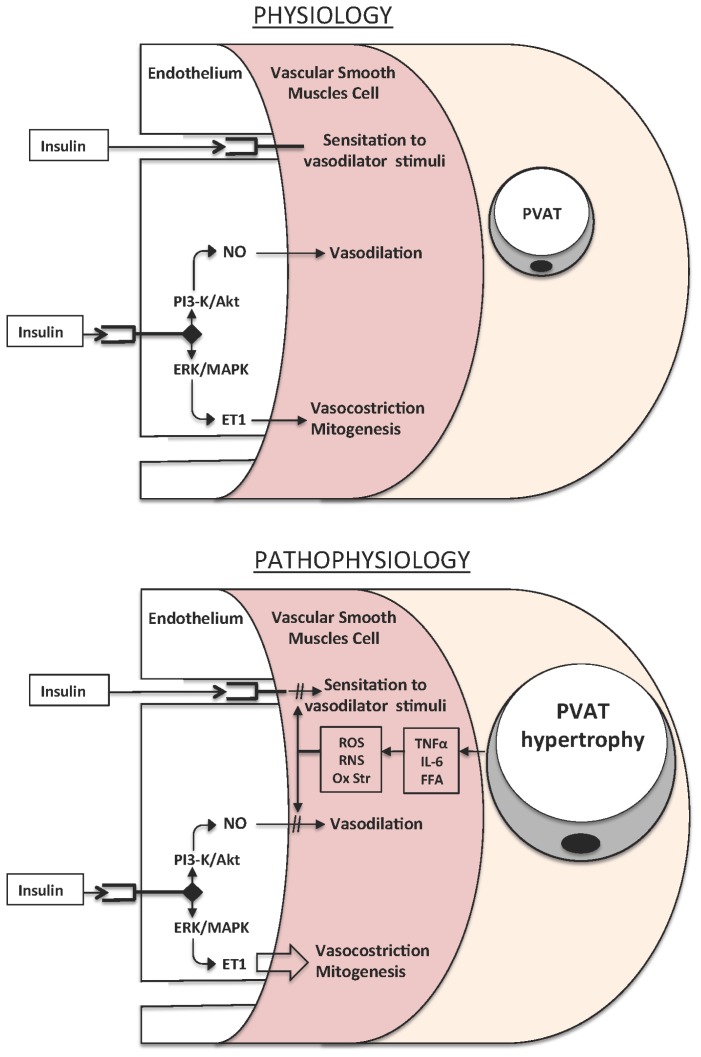
Vascular effects of insulin in the healthy state and in conditions characterized by increased oxidative stress and insulin resistance. PVAT indicates perivascular adipose tissue, all other abbreviations are spelled out in the text.

The pathology of atherosclerosis is complex and involves structural elements of the arterial wall, platelets, leukocytes and inflammatory cells, such as monocytes and macrophages [94]. The endothelium is a dynamic interface between the arterial wall and the circulating cells. Therefore, endothelial dysfunction is one of the primary causes of atherosclerosis (Figure 2). Given that the endothelium is the major source of NO in the vasculature, loss of the normal cell function can result in altered NO synthesis. Endothelium provides a constitutive supply of NO from eNOS and, under conditions such as inflammation it can also produce excess NO from the inducible isoform iNOS [95]. Diabetic complications are characterized by endothelial dysfunction and a number of studies have suggested that ROS play an important role in the pathogenesis of diabetic vasculopathy [81]. It has been documented that endothelium in diabetes fails to produce sufficient amount of NO and that blood vessels fail to relax in response to endothelium-dependent vasorelaxants (e.g., acetylcholine, bradykinin, shear stress, *etc.*) [96]. In particular, increased plasma glucose leads to increased mitochondrial formation of superoxide, a ROS that produces peroxynitrite when reacting with NO. Thus, peroxynitrite production is increased in platelets from diabetic individuals [97] and levels of nitrotyrosine in endothelial cells, myocytes and fibroblasts significantly relate to the degree of cell death [98] both in the heart of diabetic patients and in those of streptozotocin-induced diabetic rats [99].

Peroxynitrite induces cellular damage through several mechanisms, including depletion of tetrahydrobiopterin (BH4), the cofactor of eNOS for NO biosynthesis, and degradation of different biomolecules in vascular endothelium, vascular smooth muscle and myocardium. These latter changes, in turn, may lead to cardiovascular dysfunction via DNA strand breakage [100] and consequent activation of the nuclear enzyme poly(ADP-ribose) polymerase (PARP)-1 [101,102]. PARP-1 activation represents an important process in the development of vascular dysfunction both in diabetic animals and in humans [103–105], and may also contribute to the development of other diabetic complications, such as nephropathy, neuropathy and retinopathy [106]. Diabetes has been found associated with increased peroxynitrite formation both in experimental animals and in humans [107]. To further support the role of peroxynitrite/nitrotyrosine in the pathogenesis of vascular dysfunction in diabetes, neutralization of peroxynitrite with the metalloporphyrin peroxynitrite decomposition catalyst FP15 ameliorates cardiac and endothelial function in a murine model of diabetes [108]. Further evidence supports the important role of peroxynitrite in the pathogenesis of diabetic cardiomyopathy. Thus, pharmacological neutralization of peroxynitrite improves cardiac function in acute myocardial infarction, chronic ischemic heart failure and diabetic cardiomyopathy [102,103,109]. The mechanisms of cardioprotection by neutralization of peroxynitrite include defense against vascular and myocardial tyrosine nitration, PARP activation and lipid peroxidation [102,103,109]. Supplementary mechanisms of peroxynitrite-mediated diabetic cardiac dysfunction involve inhibition of myofibrillar creatine kinase [110] and succinyl-CoA:3-oxoacid CoA-transferase [111], as well as activation of metalloproteinases [100]. In addition, a role of peroxynitrite seems also present in the pathogenesis of microvascular injury underlying diabetic retinopathy [112,113], nephropathy [114] and neuropathy [115,116].

Another aspect that has recently been related to glucose-induced oxidative stress is glucose variability [117]. Thus, several studies have suggested that intermittently low or high glucose levels are even more deleterious to endothelial cell function than a steady, constant increase of glucose. Those conditions, in fact, induce endothelial cells to enter into a pro-inflammatory state, which is associated with up-regulation of various adhesion molecules and inflammatory cytokines [118]. The pathways implicated in these exacerbated cellular responses involve activation of protein kinase C (PKC), NADPH oxidases and mitochondrial oxidants.

Animal models of diabetes are associated with reduced bioavailability of NO and impaired endothelium-dependent relaxation [119]. Studies involving streptozotocin-induced diabetic rats show that diabetes-induced increase in both retinal vascular endothelial growth factor (VEGF) concentrations and lipid peroxidation could be prevented by antioxidant treatment [120]. These results suggest a major role for NO destruction by O_2_^−^ in diabetes-associated vascular dysfunction. Interestingly, eNOS knockout mice exhibit accelerated diabetic nephropathy [121], supporting a role for deficient NO production in the pathogenesis of diabetic nephropathy. Further clinical data have demonstrated that rapid glycemic swings are associated with an increased availability of oxidant products, which are deleterious for endothelial function of type 2 diabetic patients [96].

There are different potential mechanisms linking enhanced oxidative stress and vascular dysfunction in diabetes and insulin resistant states. One of these mechanisms regards the “dual” physiological effect of insulin to stimulate endothelial production of both vasodilator/antiatherogenic mediators like NO and vasoconstrictor/proatherogenic substances like endothelin (ET)-1 [122]. In the healthy state, there is a balance between these opposing forces, whereas *in situ*ations of enhanced oxidative stress and insulin resistance this balance is tilted toward a predominance of vasculotoxic forces [83]. Another mechanism relates to the loss of the physiological function exerted by insulin to increase vasodilator responsiveness and hence favor the delivery of substrates to peripheral tissues. We have extensively investigated the determinants of altered insulin stimulated vascular reactivity in insulin resistant patients with obesity by assessing the effects of insulin on vascular responses to vasodilators acting though different mechanisms. We have observed that the physiological effect of hyperinsulinemia to potentiate vasodilator reactivity has a generalized impairment in these patients. Interestingly, infusion of vitamin C during concurrent hyperinsulinemia is able to restore the facilitatory effect of insulin on vasodilator reactivity in these patients, thus confirming that oxidative stress plays a role in this abnormality [123]. We have also evaluated the possible involvement of the Rho A/Rho kinase (ROCK) pathway in the abnormal insulin signaling in obese blood vessels by use of the ROCK inhibitor fasudil. These studies have shown that Rho kinase inhibition improves the vasodilator capacity during hyperinsulinemia by quenching oxidative stress [124]. We have then assessed the effect of glucagon-like peptide 1 (GLP-1), a gut peptide that stimulates insulin secretion and sensitivity, on insulin-stimulated vascular reactivity in patients with the metabolic syndrome. This investigation has demonstrated that GLP-1 improves both endothelium-dependent and -independent vasodilation during hyperinsulinemia. In addition, when GLP-1 is given on top of vitamin C it does not further enhance the vasodilator effect of acetylcholine and sodium nitroprusside, thereby suggesting that its effect is influenced by vascular oxidative stress [125].

## 3. Oxidative Stress and Other Diabetic Complications

Type 2 diabetes is the leading cause of blindness, non-traumatic lower-limb amputation and chronic kidney disease [126,127]. Many experimental models of both types 1 and 2 diabetes exhibit increased ROS generation, triggered in large part by HG [128]. In the development of diabetes, HG triggers the overproduction of superoxide and H_2_O_2_, which, in turn, determine a decline in the antioxidant systems, directly damage many biomolecules; increase lipid peroxidation and results in insulin resistance [81]. Mullarkey *et al.* have proposed that glycated proteins enhance superoxide production and lipid peroxidation compared to non-glycated ones, thus suggesting that increased protein glycation accelerates lipid damage in diabetes [44]. Subsequent studies, however, have shown that the most important sources of ROS under hyperglycemic conditions are mitochondria and NADPH oxidases, whose overproduction of ROS causes hyperglycemia-induced damage through the following mechanisms:

1. *Activation of the polyol pathway*, probably by means of consumption of NADPH, an important scavenger of ROS [129].
2. *Increase in intracellular advanced glycation end-products (AGEs) formation*, stemming from non-enzymatic reaction of glucose and other glycating compounds with proteins [130,131].
3. *Increased expression of the receptor for AGEs and its activating ligands*: the receptor for AGE binding (RAGE) induces the production of ROS, which in turn activates the pleiotropic transcription nuclear factor NF-κB, causing multiple pathological changes in gene expression [132,133].
4. *Increased PKC activation*: PKCs are a family of at least 11 isoforms that can phosphorylate various target proteins [134]. Persistent and excessive activation of several PKC isoforms has been implicated in the decreased NO production in smooth muscle cells and has been shown to inhibit insulin-stimulated expression of eNOS in cultured endothelial cells. Activation of PKC by high glucose also induces expression VEGF, thereby enhancing permeability in vascular smooth muscle cells [135,136].
5. *Activation of the hexosamine pathway*: hyperglycemia and insulin resistance-induced excess of fatty acid oxidation contributes to the pathogenesis of diabetic complications by increasing the flux of fructose-6-phosphate into the hexosamine pathway. Fructose 6-phosphate is the substrate for the rate-limiting enzyme of the glutamine:fructose 6-phosphate amidotransferase (GFAT) pathway, which converts fructose 6-phosphate into glucosamine 6-phosphate that is in turn converted into uridine-diphosphate (UDP)-*N*-acetylglucosamine. Inhibition of GFAT may block the hyperglycemia-induced increases in the transcription of both TGF-α and TGF-β1 [46]. In addition, specific inhibitors of aldose reductase activity, AGE formation, RAGE ligand binding, PKC activation and hexosamine pathway flux may ameliorate diabetes-induced abnormalities in cell culture or animal models [137].

It has now been established that the different pathogenic mechanisms described above stem from a single hyperglycemia-induced process, the overproduction of superoxide by the mitochondrial electron-transport chain [45,138]. Thus, in cells with high intracellular glucose concentration, there is more glucose-derived pyruvate to increase the flux of electron donors (NADH and FADH2) into the electron transport chain. Coenzyme Q donates the electrons to molecular oxygen, hence leading to generation of superoxide anions. The mitochondrial isoform of the enzyme SOD, by contrast, degrades this oxygen free radical to H_2_O_2_, which is then converted to H_2_O and O_2_ by other enzymes [139]. Dynamic changes in mitochondrial morphology are associated with high glucose-induced overproduction of ROS [140].

### 3.1. Retinopathy

**Figure 3:**
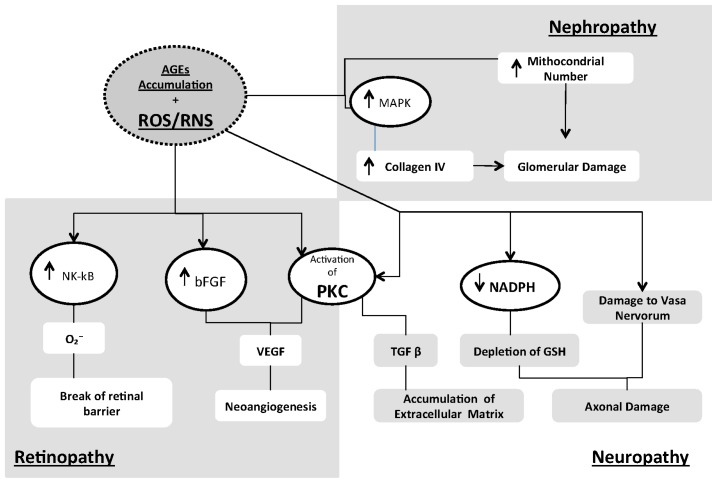
Schematic representation of the pathophysiological role of AGE accumulation and increased oxidative stress in diabetic complications like retinopathy, nephropaty and neuropathy. All abbreviations are spelled out in the text. ↑ indicates increased levels; ↓ indicates decreased levels.

Diabetic retinopathy is one of the most important causes of visual loss. It is considered a neurovascular disease with damage to retinal ganglion and glial cells [141], and it is classified as non-proliferative or proliferative retinopathy. The latter form is caused predominantly by increased angiogenesis induced by ischemia, which can lead to hemorrhages and retinal detachment. Patients with non-proliferative retinopathy usually have higher concentrations of NO that increase directly with the severity of the disease. By contrast, total antioxidant capacity has been reported to decrease directly with the progression of the disease, hence suggesting a pathogenic role of enhanced oxidative stress [142]. Oxidative stress seems to occur predominantly by inducing apoptosis of perycites, which play a central role in maintaining retinal homeostasis [143] (Figure 3). Accumulation of AGEs and consequent damage to retinal microvessels is another mechanism involved in diabetic retinopathy [143]. Thus, AGEs are known to activate the NF-κB pathway, thereby increasing the production of superoxide anions, which in turn may cause leukostatis, expression of ICAM-1 and breakages of the retinal barrier [144]. ROS can also increase PKC activity, with augmented levels of DAG, leading to endothelial dysfunction and increased vascular permeability due to higher production of VEGF. Basic fibroblast growth factors (bFGF) synthesized in a chronic hypoxic environment or under conditions of increased oxidative stress have also been involved in the neoangiogenic process [145].

### 3.2. Nephropathy

Diabetic nephropathy is the leading cause of end-stage renal disease (ESRD) in Western countries. It is characterized by accumulation of mesangial cells, collagen IV, fibronectin and laminin. Accumulation of these substances activates the oxidative stress cascade and results in overproduction of ROS. As in the eye, mesangial cells are essential to preserve normal function also in the kidney [143] (Figure 3). By contrast, in diabetic patients accumulation of AGEs can induce TGF-β synthesis, thereby leading to extracellular fibrosis. Studies have demonstrated a role for the Src pathway in this process. Src is a non-receptor tyrosine kinase activated by receptor tyrosine kinases such as EGFR. Different stimuli, such as ROS or high glucose levels, can induce Src-dependent EGFR transactivation, thus resulting in increased synthesis of MAPK and collagen IV [146]. Glycation of some proteins, such as laminin and collagen IV, can also alter vascular permeability to albumin, thus contributing to renal damage. In addition, high glucose may decrease the expression of mitochondrial anti-oxidants, such as manganese superoxide dismutase, with consequent impairment of the electron chain and overproduction of ROS. Oxidative stress, in turn, is a stimulus for the synthesis of mitochondrial DNA. Because mitochondria are the main intracellular source of ROS, an increase in their number amplifies cell exposure to ROS and increases kidney damage [147].

### 3.3. Neuropathy

Diabetic neuropathy affects almost 30% to 50% of patients with diabetes (Figure 3). AGEs have been involved in the pathophysiology of this complication due to their ability to inhibit axonal regeneration by induction of myelin and microfilament changes [148]. Activation of the polyol pathway can also contribute to oxidative stress, by causing NADPH depletion and consequent decrease in intracellular glutathione. Another factor involved in diabetic neuropathy is increased DNA damage stemming from enhanced production of NO; the resulting activation of poly ADP ribose polymerase (PARP) lowers NADPH and is a stimulus for secretion of inflammatory mediators [146]. Finally, a pathogenic role of PKC has been postulated, given that high glucose levels increase diacylglycerol concentrations, thereby leading to stimulation of NF-κB and TGF-β and to increased deposition of extracellular matrix [149].

## 4. Antioxidant Therapy

Pathophysiological evidence and observational studies have provided the rational basis for intervention trials performed to assess whether manipulation of the oxidant-antioxidant balance could prevent and/or improve complications of diabetes. These human intervention trials have predominantly attempted to increase antioxidant defense by antioxidant supplementation. Improved insulin sensitivity and glucose tolerance have been observed with lipoic acid [150–153], NAC [154], vitamin E [155–158], and vitamin C [159]. These results have been mainly observed in small-sized, short-term trials and, frequently, other studies using the same agents in similar populations have demonstrated no measurable effects of [160–163]. Late diabetes complications also do not seem to be positively affected by antioxidant therapy [164]. Also, despite some evidence supporting the ability of antioxidants to improve insulin action, it is not currently recommended to supplement antioxidants in patients with impaired insulin action, like type 2 diabetes [165]; this is also because of some evidence suggesting potential harm, including increase in all-cause mortality, with vitamin E, carotene, selenium, and other antioxidant supplementation [166–168].

It is difficult to believe that these disappointing results definitely rule out a mechanistic role for ROS/RNS in the induction of cardiovascular complication in human diabetes. More likely, the extremely complex oxidant-antioxidant systems and their intricate, occasionally opposing roles in physiology and pathophysiology render currently existing interventions aimed at manipulating the oxidant-antioxidant balance clinically ineffective. This could be either because supplemented antioxidant is buffered by other components of the system that are in equilibrium with it, because excessive antioxidants can be converted to pro-oxidants, or because a specific antioxidant simply does not affect all the mechanisms responsible for excessive oxidant generation. It is generally recommended to consume sufficient amounts of naturally occurring dietary antioxidants by balanced eating habits [165]. It is possible to speculate that consuming diets high in antioxidants can exert beneficial effects by providing balanced antioxidant mix or that they include novel, yet uncharacterized, antioxidants. The most recent studies also underline the importance of treating early hyperglycemia-induced oxidative stress, because the redox unbalance can determine epigenetic changes that persist during normoglycemia (metabolic memory) [169,170]. Addressing all these possibilities by well-designed clinical studies will further our current understanding of the role of ROS/RNS in diabetes and aid the development of therapies to prevent diabetic complications and their heavy toll on human health.

## 5. Conclusions

A large body of evidence suggests that oxidative stress has a key role in the pathogenesis of diabetes and its complications. Clearly, both insulin resistance and β-cell dysfunction, two central events in the pathophysiology of type 2 diabetes, are linked to a redox unbalance. At the same time, oxidative stress has been implicated in the pathogenesis of diabetes-related vascular complications. Oxidative stress in diabetic vascular disease contributes markedly to endothelial and smooth muscle dysfunction. It is mainly caused by an imbalance between the activity of endogenous pro-oxidative enzymes (such as NADPH oxidase, xanthine oxidase, and mitochondrial respiratory chain) and antioxidative enzymes (such as superoxide dismutase, glutathione peroxidase, heme oxygenase, and catalase) resulting in a production of ROS that exceeds the available antioxidant defense systems. As a consequence, bioactivity of NO, a paracrine factor that controls vascular tone, inhibits platelet function, prevents adhesion of leukocytes, and reduces the proliferation of the intima (anti-atherosclerotic mechanism), is reduced. A dominant mechanism reducing bioavailability of vascular NO relates to its rapid oxidative inactivation by the ROS. There is also evidence that persisting oxidative stress renders eNOS dysfunctional and unable to produce NO. In addition to reduced NO bioavailability, oxidative stress at the vascular smooth muscle cell level contributes to impaired insulin signaling and consequent vascular dysfunction. All these data support the concept that antioxidant therapy may be of great interest in diabetes and related conditions. To test this hypothesis, several studies have been conducted, but their conflicting results underscore the need of further investigation to better understand whether antioxidant treatment may be helpful in the prevention of diabetes and its complications.
